# Single Incision Laparoscopic Surgery: Feasibility of the Direct Fascial Puncture Technique Without Working Trocars

**DOI:** 10.7759/cureus.10742

**Published:** 2020-09-30

**Authors:** Shamir O Cawich, Giovanni Dapri, Patrick Fa Si Oen, Dexter Thomas, Vijay Naraynsingh

**Affiliations:** 1 Surgery, University of the West Indies, St. Augustine, TTO; 2 Surgery, International School of Reduced Scar Laparoscopy, Brussels, BEL; 3 Surgery, Princess Margaret Hospital, Curacao, CUW; 4 Surgery, West Shore Hospital, St. Augustine, TTO; 5 Surgery, Medical Associates Hospital, St. Joseph, TTO

**Keywords:** cholecystectomy, laparoscopic, sils, direct, puncture, fascia, technique

## Abstract

Introduction

As single-incision laparoscopic surgery (SILS) became popular, many access platforms and techniques emerged. When we initially described the direct fascial puncture (DFP) technique, many suggested it was not practical for three reasons: (1) increased hernia formation, (2) inability to complete operations without instrument changes and (3) insurmountable instrument drag. This study sought to determine whether the technique was a feasible approach by evaluating the outcomes with DFP-SILS in a single surgeon unit.

Methods

This was a retrospective audit of all consecutive patients who had unselected SILS operations by a single surgeon. For the DFP-SILS operation, a single optical trocar was used at the umbilicus, a second was rail-roaded beside the optical trocar and a third was directly passed across the fascia at the left-lateral extent of the skin wound. We recorded the number of conversions or failed operations and examined the patients routinely after operation to evaluate for incisional herniae.

Results

There were 50 DFP-SILS operations performed: 37 cholecystectomies, 12 appendectomies and one jejunal resection. The operations were successful in all cases with no conversions or mortality recorded. One patient (2%) developed a superficial surgical site infection after SILS-DFP appendectomy. The therapeutic outcomes were comparable to existing series of multi-port laparoscopy. There were no incisional herniae detected.

Conclusion

Even in the resource-poor setting, SILS operations are feasible and safe using the DFP technique. The theoretic concerns have not been realized in clinical practice.

## Introduction

Each port used in conventional laparoscopy has the potential to cause pain, bleeding, local nerve irritation, incisional herniae and reduced aesthetics [1-2]. This fueled the development of single incision laparoscopic surgery (SILS) in the late 20th century. There have been several modifications of SILS techniques since Navarra et al. reported the first SILS operation in 1997 [3].

Similarly, we have modified our technique since our first SILS operation [4]. The direct fascial puncture (DFP) technique was first performed in 2011 and has been described in detail in prior reports [5]. When we submitted reports of our early experience using the DFP technique for publication, many reviewers declined to publish the manuscript citing three reasons this technique was impractical.

First, they argued that most operations required multiple instrument changes and this technique would not allow the instrument passed directly across the fascia to be changed because the original track would not be found once the first instrument was removed. Second, it was suggested that there would be excess drag applied to working instruments passed directly across the fascia since there was no port to facilitate instrument gliding and protect it from contact with surrounding tissues. Finally, it was suggested that the incidence of incisional herniae would rise because the fascial puncture was in or near the midline. The resultant “fascial tattering” from a direct puncture would result in an attenuated midline fascial bridge and subsequent incisional herniae.

We found the DFP technique feasible in our hands but did not have data to support this. Therefore we sought to document our experience with the DFP-SILS technique. The primary aim of this study was to determine the feasibility of completing SILS operations using the DFP technique. A secondary aim was to determine the short-term incidence of incisional herniae in these patients.

## Materials and methods

Our first DFP-SILS operation was performed in 2011 and has already been described in detail in prior reports [5]. In summary, we noticed that “instrument clashes” did not involve the instruments but occurred between the port platforms and this prompted our move to the SILS-DFP technique. Here, a 2-cm incision was created across the umbilicus and the skin undermined to maximize fascial exposure [5]. An 11-cm incision was created in the fascia at the left side of the fascial window and a purse string polypropylene suture was inserted at the margins of the fascial incision. The purse string suture was tightened around a 10-mm port to achieve a seal and create a pneumoperitoneum. A 5-mm trocar introducer was then used to puncture the fascia at the right side of the wound [5]. The introducer was withdrawn and an electrocautery hook was immediately advanced across the fascial tract. The purse string suture was then relaxed and a 5-mm instrument was then passed beside the optical port, allowing the purse string suture to encircle both instruments maintaining the pneumoperitoneum. The electrocautery hook was the instrument we most commonly used in our hands and was not removed until the operation was completed.

The local institutional review board granted permission to collect data from all consecutive patients who underwent SILS operations. All procedures followed were in accordance with the ethical standards of the Helsinki Declaration of 1975, as revised in 2000. We reviewed hospital registers to identify all patients who had DFP-SILS operations carried out by a single surgical team from May 1, 2012 to May 1, 2015. This period was chosen to allow a minimum of five-year follow-up to determine the incidence of incisional herniae. Hospital records were retrieved and the following data were extracted retrospectively: patient demographics, indications for operation, operative details, conversions, morbidity, mortality and incisional herniae. Descriptive statistics were generated using SPSS version 12.0 (SPSS Inc., Chicago, USA).

Any procedure using a laparoscopic approach in which all instruments were placed across one incision at the umbilicus was considered a SILS operation. Any procedure where the working instruments were introduced across a direct fascial puncture without the use of trocars was considered a DFP-SILS operation. A conversion was considered to be any procedure in which an additional incision was required separate from the umbilical incision - whether for open access, to place an additional port or introduce other devices to assist in the operation. In the event a formal SILS platform or an extra trocar was inserted into the fascia through the single incision, we also considered that a conversion. Any operation performed in an operating room on anesthetized patients requiring <24 hours hospitalization was considered an ambulatory procedure. This was the standardized definition used by the United States Planning and Research Cooperative System committee [6].

Complications were graded according to their severity using the standardized grading system for surgical complications proposed by Clavien et al. [7]. Grade I-II complications were considered mild and grade III-IV complications as major morbidity. Post-operative mortality was defined as death from any cause within 30 days of operation. We routinely evaluated patients with detailed clinical examination six months after their operation. Clinical examinations were used to determine the integrity of the umbilical wound and to detect incisional herniae. Telephone follow-up was performed to identify symptoms that may indicate delayed diagnoses of incisional herniae. In these instances, patients were asked to return to clinic for detailed physical examination.

## Results

Over the study period, the DFP-SILS technique was used to perform 37 cholecystectomies, 12 appendectomies and one jejunal resection for a carcinoid. No conversions were required.

Elective cholecystectomies were performed in 37 women at a mean age of 36.2 years (SD 8.9; Range 18-60) and a mean body mass index of 30 (SD 2.6; Range 24-35). No operative cholangiograms were performed. These operations were completed in a mean of 64.9 minutes (SD 19.3; Range 42-150) with 64.1 ml mean blood loss (SD 67.8; Range 20-350), no overall morbidity and no incisional herniae. There were no bile duct injuries or deaths recorded in this series. A total of 31 patients (84%) were discharged from hospital within 24 hours.

Appendectomies were performed emergently in 12 patients (nine women and three men) at a mean age of 30.1 years (SD 9.5; Range 18-42) and a mean body mass index of 28.3 (SD 1.4; Range 26-30.4). The average operating time for SILS-DFP appendectomies was 40 minutes (Range 25-70; SD 16.8). One patient developed a superficial surgical site infection (Grade I) that resolved after a course of antibiotics. There were no conversions and no incisional herniae. There was one small bowel resection performed in this series. In this case, there were no complications, conversions or incisional herniae reported.

Although the case mix was heterogenous, we included all SILS operations because we set out to evaluate the feasibility of the DFP technique, specific to the three concerns raised. Overall, SILS operations were being performed using the DFP technique with no conversions, acceptable complication rates (2% minor morbidity) and no mortality.

## Discussion

The first SILS operation in the Caribbean was an elective cholecystectomy done in 2009 [4]. Over the subsequent years, our experience increased and more complex cases were attempted, including sleeve gastrectomies, colectomies and cholecystectomies for acute cholecystitis [8,9]. With the accumulating experience, we also modified our technique. Although reviewers initially suggested that the SILS-DFP technique was impractical, the theoretic concerns that would render this technique unfeasible were not seen in clinical practice.

We agree that instrument changes are required to complete most operations. We also agree that the instrument passed directly across the fascia cannot be changed easily because the original track is difficult to re-cannulate once the first instrument is removed. However, we overcame this limitation by carefully selecting the instrument passed directly across the fascia using one that would remain in-situ until the operation was completed. Initially, we rationalized that the laparoscope was the instrument that would always be required. Therefore, we attempted to pass the scope directly across the fascia but this was not practical because the scope would need to be removed occasionally for cleaning. Therefore, we chose to pass the second most commonly used instrument directly across the fascia. In our hands, this was the electrocautery hook that we routinely used to skeletonize the structures in Calot’s triangle and to dissect the gallbladder from the hepatic bed [5]. Our practice did not involve the routine use of ultrasonic dissectors, which were not universally available in this resource poor setting [9,10]. Change of this instrument was not required in any case in our series. Even in instances where the active end of the instrument became heavily laden with coagulum, we did not remove the instrument. Instead, a gauze swab was passed through the visual port and used to clean the instrument tip intra-corporeally with good effect.

The second theoretic concern raised was that the absence of a working port to facilitate instrument gliding would render the instrument difficult to use because excessive drag would be applied to the instrument when it contacted surrounding tissues. We were prepared to lubricate the instrument by pouring sterile saline into the wound to reduce drag, but that was unnecessary because we did not encounter the problem in clinical practice. We found the instrument relatively easy to handle. Although we concede that this is subjective, we could not find an objective way to evaluate the effect of drag applied to the working instruments. Moreover, the absence of working trocars improved ergonomics by eliminating collisions between bulky trocar heads. Consequently, we found the instruments easy to handle compared to “conventional SILS.”

The third theoretic limitation to the DFP technique was the potential to increase the rate of incisional hernia formation. In our initial reports, we suggested that the 5-mm defect left at the end of the DFP technique did not require closure with sutures [5]. But opponents suggested that a direct puncture would leave the fascia tattered, thus attenuating the fascial bridge at the midline umbilical incision. This, they argued, would predispose to incisional herniae. However, we have not detected any herniae after a minimum of five years follow-up, despite our practice of routinely omitting any attempts to close the fascial puncture wound. Admittedly, these are short-term results and the potential for incisional hernia will persist for several years. Therefore, we concede that these data do not completely settle this concern and that it would require physical re-evaluation of the patients after a longer interval.

From a theoretic point of view, we suggest that there should be no difference between the fascial defect created by a standard 5-mm trocar and that from direct fascial puncture with a 5-mm instrument. Most surgeons do not routinely close the 5-mm trocar sites in multiport laparoscopy, even when placed in midline locations [11]. We understand the theoretic concern that the fasica is inherently weak at the midline [12]. However, in the DFP technique the visual trocar is passed in the midline and this is routinely closed with sutures. The direct fasical puncture is then placed off-center to the extreme left of the skin incision in order to reduce instrument clashes [5]. Therefore, the rectus abdominis muscle would buttress the fascial puncture site, reducing the relative risk of port-site herniae.

These data support the DFP-SILS technique as being feasible despite the theoretic concerns. The therapeutic outcomes of SILS operations using the DFP technique were good. Table 1 demonstrates that the operative time, morbidity and mortality for SILS operations using this technique were comparable to those in existing reports of multi-port laparoscopy for elective operations in the Caribbean [13-21]. The results are comparable to existing standards with multiport laparoscopy in the Caribbean region.

**Table 1:** A comparison of outcomes with SILS-DFP and conventional multi-port laparoscopy for elective operations in the Caribbean NR: Not recorded; SSI: Surgical site infection; SILS: Single-incision laparoscopic surgery; DFP: Direct fascial puncture. * Elective series in cohort with sickle cell disease

Parameter	SILS-DFP	Dan et al. [19]	Dan et al. [20] *	Mitchell et al. [13]	Plummer et al. [16] *	Cawich et al. [17]	McFarlane et al. [14]	Cawich et al. [18]	Cawich et al. [21]
Elective Laparoscopic Cholecystectomy									
Surgery time (min)	65 ± 19.3	34	27.9	109	108	83	85	94 ± 21.7	-
Overall morbidity	0	1.5%	21.1%	11.9%	37.5%	8%	14.4%	8.1%	-
Bile duct injury	0	0	0	1%	0	0	0.5%	0	-
Minor Bile leaks	0	0.2%	NR	6.9%	NR	NR	0.5%	1.0%	-
Haemorrhage	0	0.5%	5.3%	1%	NR	4.2%	2.6%	4.0%	-
Port site hernia	0	1.0%	NR	NR	NR	0	0	0	-
Mortality	0	0	0	0	6.3%	0	0	0	-
SSI	0	0.4%	0	3%	6.3%	0	NR	0	-
Conversions	0	0.7%	0	5.9%	25%	7.7%	6.1%	2.0%	-
Admission >24 Hrs	16%	48%	NR	100%	NR	8%	30%	55.4%	-
Laparoscopic Appendectomy									
Surgery time (min)	40 ± 16.8	-	-	-	-	-	-	-	50 ± 18.4
Overall morbidity	8.3%	-	-	-	-	-	-	-	4.2%
Conversion	0	-	-	-	-	-	-	-	0.5%
Mortality	0	-	-	-	-	-	-	-	0
SSI	8.3%	-	-	-	-	-	-	-	2.4%

We acknowledge that there are limitations to this study. The first limitation is the use of clinical examination to detect incisional herniae. We acknowledge that clinical examination to detect incisional herniae may not be as accurate as imaging studies, introduces subjective bias and may be challenging as the average patient body mass index in this study was 28.3. A second limitation was that the study evaluated outcomes of the SILS-DFP technique by a single surgical service in one institution. We acknowledge that the results may not easily be reproducible in the wider surgical community by multiple surgeons with varied levels of experience in multiport laparoscopy. Therefore, it would be prudent to design a future trial to evaluate the outcomes of this technique in a multi-centre, multi-surgeon trial with larger case volumes.

## Conclusions

Theoretic concerns that made the DFP technique for SILS impractical have not been realized in clinical practice. The technique has been used in the Caribbean setting with good outcomes that are comparable to existing reports of conventional multi-port laparoscopy from the region. The technique provides a good blend of ergonomics, effectiveness and cost-containment.
